# Conceitos atuais em fraturas intra-articulares do calcâneo

**DOI:** 10.1055/s-0045-1809887

**Published:** 2025-07-29

**Authors:** Rafael Barban Sposeto, Germán Matías-Joannas, Alexandre Leme Godoy-Santos

**Affiliations:** 1 Departamento de Ortopedia e Traumatologia, Instituto de Ortopedia, Hospital das Clínicas da Universidade de São Paulo, São Paulo, SP, Brasil.; 2 Divisão de Pé e Tornozelo, Centro de Estudios y Práctica Profesional (CEPP), Instituto Dupuytren, Buenos Aires, Argentina.

**Keywords:** articulação talocalcânea, calcâneo, fixação interna de fraturas, fraturas intra-articulares, fraturas ósseas, ossos do tarso, calcaneus, fracture fixation, internal, fractures, bone, intra-articular fractures, subtalar joint, tarsal bones

## Abstract

As fraturas intra-articulares do calcâneo são lesões significativas do sistema locomotor, muitas vezes levando a deficiências permanentes na mecânica do pé, com repercussões ocupacionais, sociais e financeiras importantes. A primeira técnica de diagnóstico por imagem é a radiografia; no entanto, a tomografia computadorizada é crucial para entender a anatomia tridimensional da fratura e facilitar o planejamento cirúrgico.

O tratamento de fraturas intra-articulares do calcâneo ainda é debatido, e a literatura apoia diversas abordagens para tipos de fraturas semelhantes. Hoje, o padrão-ouro para o tratamento de fraturas da articulação subtalar é a intervenção cirúrgica. Entre as duas técnicas mais comuns, a abordagem lateral estendida e a abordagem do seio do tarso produzem desfechos funcionais comparáveis, embora a última esteja associada a menos complicações.

Este artigo discute o diagnóstico, a classificação e o tratamento das fraturas intra-articulares do calcâneo, com foco nas abordagens do seio do tarso e do “L” lateral estendido, bem como as técnicas de fixação aplicáveis a cada tipo de fratura.

## Abstract (en)

Intra-articular calcaneal fractures are significant injuries to the locomotor system, often leading to lifelong impairments in foot mechanics with substantial occupational, social, and financial repercussions. The initial imaging diagnosis relies on radiography; however, computed tomography is crucial for understanding the three-dimensional anatomy of the fracture and facilitating surgical planning.

The treatment of intra-articular calcaneal fractures remains a subject of debate, with literature supporting diverse approaches for similar fracture types. Currently, the gold standard for managing subtalar joint fractures is surgical intervention. Among the two most common techniques, the extended lateral and the sinus tarsi approaches yield comparable functional outcomes, though the latter is associated with fewer complications.

The present article discusses the diagnosis, classification, and treatment of intra-articular calcaneal fractures, focusing on the sinus tarsi and extended lateral “L” approaches, as well as the fixation techniques applicable to each fracture type.

## Introdução

O calcâneo é o maior osso do tarso. Entre os ossos do pé, o calcâneo é o mais acometido por lesões (60%), representando 1 a 2% de todas as fraturas do corpo. Além disso, 75% das fraturas do calcâneo são intra-articulares. 1 2 Estas são algumas das fraturas articulares de tratamento mais desafiador, muitas vezes produzindo desfechos insatisfatórios para pacientes e médicos. 3

A maioria das fraturas do calcâneo é decorrente de traumas de alta energia e ocorre principalmente em pacientes jovens e ativos. 4

Há controvérsia em relação ao tratamento ideal dessas fraturas. As abordagens cirúrgicas e não cirúrgicas têm suas vantagens e desvantagens. No entanto, ao longo do tempo, o tratamento cirúrgico surgiu como a abordagem preferida para fraturas do calcâneo. 2 4 5 6 7

A literatura demonstra que a redução anatômica e a fixação interna proporcionam os melhores desfechos em termos de recuperação rápida e restauração precoce da função da articulação subtalar. 7 8 O padrão-ouro terapêutico deve incluir redução anatômica da articulação subtalar, restauração da largura, do alinhamento e do comprimento normais do calcâneo e estabilização com fixação rígida. 5

## Anatomia

O calcâneo, juntamente com o tálus, forma o retropé. Esse osso apresenta quatro superfícies articulares: uma articulação calcaneocuboide e uma subtalar, que se divide em facetas anterior, média e posterior. A faceta subtalar posterior é a maior e mais importante para suporte de carga durante a marcha; tem formato convexo e orientação distal e lateral em um ângulo de 45° em relação ao plano sagital. 9 A faceta média está localizada na superfície superior do sustentáculo do tálus, anterior e medial à faceta posterior. A faceta anterior, a menor, está no aspecto anterior do calcâneo, lateral ao sustentáculo do tálus. 9 10

A superfície medial do calcâneo tem córtex espesso que suporta sua projeção medial, o sustentáculo do tálus. Essa estrutura dá estabilidade à cabeça e ao colo do tálus, tendo papel fundamental na manutenção do alinhamento do retropé. Esta superfície é um sítio de inserção para o componente tibiocalcâneo dos ligamentos deltoide e calcaneonavicular plantar. 10

## Mecanismo de Trauma

Embora as fraturas do calcâneo possam ser provocadas por doenças, como tumores ou lesões por estresse, a maioria dos casos é causada por trauma axial de alta energia que afeta as articulações do calcâneo. 2 11 12

As fraturas intra-articulares do calcâneo são decorrentes de forças axiais que rompem as articulações subtalar e calcaneocuboide, bem como o corpo do calcâneo e os tecidos moles circundantes. 4 13 O padrão de fratura resultante depende da direção e intensidade da força, da posição do pé no momento da lesão e da qualidade óssea do paciente. 1

As forças axiais comprimem o calcâneo contra o tálus, e o processo talar lateral do tálus impacta o córtex calcâneo no ângulo de Gissane. Como o eixo de carga do tálus é mais medial do que o do calcâneo, há um momento de eversão, produzindo a linha de fratura primária. 1

Esta linha de fratura primária divide o calcâneo em sentido coronal em um fragmento anteromedial e um posterolateral. O fragmento anteromedial, que inclui o sustentáculo do tálus e a faceta subtalar média, continua congruente com o tálus. Por essa relação anatômica consistente, este fragmento—denominado “fragmento constante”—é uma referência importante para a redução. 2 14

O valgo do retropé no momento do trauma gera uma linha primária mais lateral e um fragmento anteromedial maior, enquanto o varo do retropé provoca uma linha mais medial e um fragmento menor, complicando a redução e a fixação durante a cirurgia. 1

À medida que a energia do trauma se dissipa, as linhas de fratura secundárias criam cominuição no corpo do calcâneo e nas articulações subtalar e calcaneocuboide. 7 14 15 Dentre as deformidades típicas, estão alinhamento em varo da tuberosidade calcânea, depressão da articulação subtalar posterior e espessamento da parede lateral, com deslocamento proximal da tuberosidade e reduções do *pitch* do calcâneo e do ângulo de Böhler. 15

O varo da tuberosidade resultante é uma composição do movimento deste segmento nos três planos. A translação lateral da tuberosidade calcânea em conjunto com o movimento rotacional medial causa varo do eixo coronal da tuberosidade. 1

## Exame Físico

As fraturas do calcâneo são frequentemente associadas a lesões em outras regiões musculoesqueléticas e sistemas corporais. Uma avaliação abrangente dos pacientes, seguindo os protocolos de Suporte Avançado de Vida em Trauma ( *Advanced Trauma Life Support* , ATLS, em inglês), é essencial. As lesões mais associadas são as fraturas do pilão tibial, platô tibial e coluna vertebral. 4

Durante o exame do pé, é crucial avaliar a dor e as deformidades no retropé, no mediopé e no antepé, assim como lesões cutâneas, déficits sensoriais, pulsos e perfusão. O diagnóstico de síndrome compartimental neste estágio é vital; a síndrome se manifesta como edema tenso, dor não responsiva a analgésicos potentes, dor intensa durante o movimento dos dedos, déficits de perfusão e parestesia. 2

Os achados clínicos comuns em fraturas do calcâneo incluem equimoses plantares, dor, edema, deformidades do retropé e dificuldade na sustentação do peso. A integridade do tecido mole é essencial para determinar o momento de realização da cirurgia e seu planejamento. A presença de flictenas geralmente indica intervenção cirúrgica tardia. De modo geral, a cirurgia é programada após a redução do edema, quando são observadas rugas na pele e cicatrização das flictenas. 1

## Técnicas de Diagnóstico por Imagem

### Avaliação Radiográfica

A radiografia é a principal modalidade de imagem para o diagnóstico de fraturas do calcâneo e deve incluir projeções em perfil, anteroposterior e oblíqua do pé e projeções axiais do calcâneo. 16

**Fig. 1:**
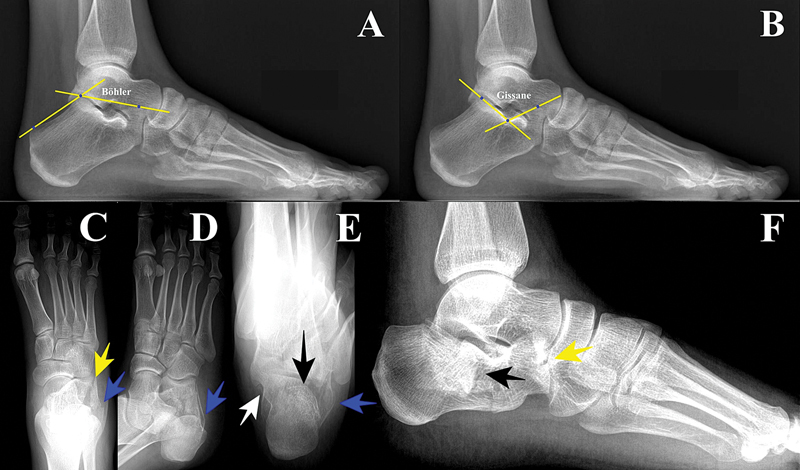
Avaliação radiográfica. ( **A** ) Ângulo de Böhler no pé normal (valores entre 20–40°). É formado pela intersecção das linhas que conectam o processo anterior, a articulação subtalar posterior e a tuberosidade calcânea. ( **B** ) Ângulo de Gissane no pé normal (valores entre 95–105°). É formado pela linha da faceta posterior e pela linha do sulco até a porção mais superior do processo calcâneo anterior. ( **C** ) Incidência anteroposterior, com fratura do calcâneo. ( **D** ) Incidência oblíqua, com fratura do calcâneo. ( **E** ) Incidência axial, com fratura do calcâneo. ( **F** ) Incidência lateral, com fratura do calcâneo. Seta amarela – fratura articular calcaneocuboide. Seta azul – espessamento da parede lateral. Seta preta – depressão da articulação subtalar posterior. Seta branca – fragmento constante.

As radiografias em perfil são usadas para medida dos ângulos de Böhler e Gissane. O ângulo de Böhler é formado pela intersecção de linhas que conectam o processo anterior, a articulação subtalar posterior e a tuberosidade calcânea. Os valores normais variam de 20 a 40°. 17 O ângulo de Gissane, medido a partir da parede lateral do calcâneo, varia entre 95° e 105° 18 ( Fig. 1 ).

A projeção anteroposterior permite a melhor visualização da articulação calcaneocuboide e do espessamento da parede lateral. As projeções oblíquas mostram o deslocamento da articulação calcaneocuboide e da tuberosidade em relação à parede lateral. As radiografias axiais revelam espessamento lateromedial, desvio em varo ou valgo e desalinhamento da articulação subtalar posterior ( Fig. 1 ).

O ajuste durante a redução dos ângulos de Böhler e Gissane, em especial do primeiro, restaura o formato do corpo do calcâneo e do retropé, proporcionando melhores desfechos funcionais. 4 12 17

A incidência de Broden complementa a avaliação radiográfica. 4 Com o paciente em decúbito dorsal e o tornozelo em posição neutra, a perna é rotacionada internamente em 30 a 40°. As imagens são obtidas em ângulos cefálicos de 40°, 30°, 20° e 10°, centralizados no maléolo lateral, para visualização detalhada da articulação subtalar posterior. No período intraoperatório, essas projeções são particularmente úteis para avaliação da redução da articulação. 4 19

### Tomografia Computadorizada

**Fig. 2:**
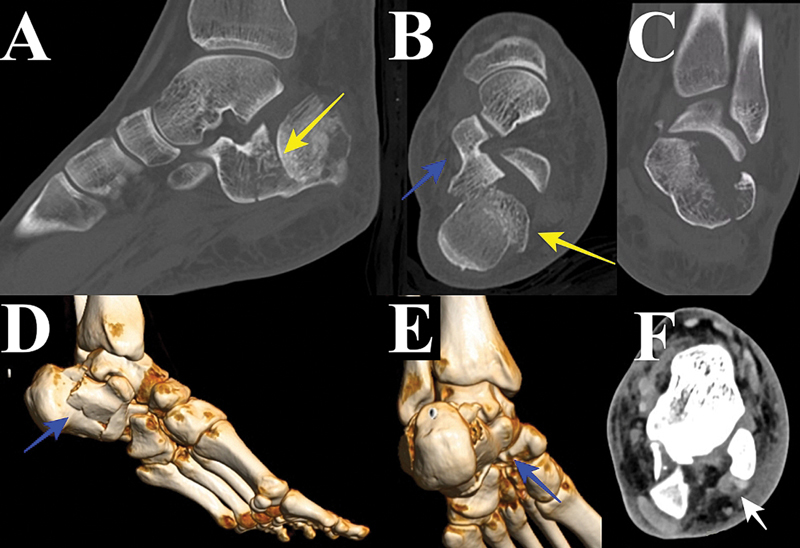
Avaliação tomográfica. ( **A** ) Tomografia computadorizada (TC) sagital. Depressão da articulação subtalar. ( **B** ) TC axial. Seta azul – fragmento constante; seta amarela – depressão da articulação subtalar. ( **C** ) TC coronal. Aumento da parede lateral e incongruência da articulação subtalar. ( **D** ) TC com reconstrução tridimensional. Seta azul – fragmento constante. ( **E** ) Reconstrução 3D por TC. Seta azul – fragmento constante. Observe o deslocamento rotacional da tuberosidade, com translação lateral. ( **F** ) Janela axial de TC de tecido mole. Seta branca – tendões fibulares.

A tomografia computadorizada (TC) melhora o diagnóstico, a classificação e o prognóstico das fraturas, 3 14 15 particularmente na identificação de lesões associadas e na compreensão do deslocamento tridimensional da fratura para planejamento cirúrgico 16 20 ( Fig. 2 ).

Como um estudo seccional submilimétrico, a TC proporciona melhor visualização do deslocamento articular, auxiliando a decisão terapêutica. Além disso, as reconstruções por TC permitem melhor compreensão da anatomia da fratura, auxiliando o planejamento cirúrgico e possibilitando a escolha de manobras de redução e tipos de fixação.

### Ressonância Magnética

A ressonância magnética (RM), embora de menor disponibilidade e maior custo, é valiosa para o diagnóstico de fraturas ocultas e patológicas (por exemplo, tumores, fraturas por estresse) bem como de lesões associadas de tecidos moles. 21

## Classificação

Existem diversas classificações para fraturas do calcâneo.

Böhler foi o primeiro a apresentar uma classificação abrangente das fraturas do calcâneo.

Em 1952, Essex-Lopresti descreveu um novo sistema que classificava as fraturas do calcâneo em dois grupos com base no mecanismo de lesão: fraturas do tipo língua e fraturas de depressão articular. 22

Em 1989, Rammelt e Zwipp 1 introduziram um sistema de classificação de 12 pontos para fraturas do calcâneo, incorporando fatores como o número de superfícies articulares acometidas, os fragmentos principais da fratura, a extensão do trauma do tecido mole e as fraturas associadas em ossos vizinhos.

Em 1993, Sanders et al. 3 publicaram uma série de 132 fraturas calcâneas com luxação e propuseram um sistema de classificação baseado em TC. Este sistema é baseado em cortes coronais em direção oblíqua, avaliando no mesmo corte a faceta posterior e sustentáculo talar, identificando três linhas de fratura, A, B e C (de lateral para medial).

- As fraturas do tipo 1 não apresentam desvio, independentemente do número de linhas de fratura.
- As fraturas do tipo 2 têm 2 fragmentos (uma única linha de fratura) e são categorizadas como 2a, 2b ou 2c, dependendo da localização da linha de fratura primária.
- As fraturas do tipo 3 têm 3 fragmentos (2 linhas de fratura), com um fragmento central deprimido, e são classificadas de forma semelhante em 3ab, 3bc ou 3ac.
- As fraturas do tipo 4 têm 4 ou mais fragmentos com cominuição significativa. 3

## Tratamento das Fraturas Articulares do Calcâneo

De modo geral, o tratamento cirúrgico proporciona desfechos funcionais superiores em fraturas com desvio da articulação subtalar, independentemente da classificação. 23 24 Nos casos de osteoartrite subtalar pós-traumática, os desfechos da artrodese são melhores se o tratamento inicial da fratura aguda for cirúrgico, com restabelecimento prévio do alinhamento do corpo e da articulação do calcâneo. 24

Considerações especiais, como doenças vasculares, tabagismo, diabetes, idade avançada, doenças sistêmicas, estado geral limítrofe e lesões extensas de tecidos moles, devem orientar a tomada de decisão cirúrgica. O tratamento cirúrgico é indicado quando o desvio da articulação subtalar posterior excede 2 mm, como definido por Sanders. 3 14 15

Seguindo os princípios AO ( *Arbeitsgemeinschaft für Osteosynthesefragen* , AO, em alemão), a redução anatômica e a estabilidade absoluta devem ser prioridades em regiões articulares. 25 A visualização direta dos componentes articulares durante a redução aberta é o método preferido para atingir esses objetivos.

O alinhamento do corpo do calcâneo é essencial para restaurar as relações funcionais no retropé e Mediopé. Os objetivos cirúrgicos incluem corrigir o ângulo de Böhler e a altura, o comprimento e a morfologia do calcâneo. 17 26 No plano sagital, é realizado o reposicionamento posterior e plantar da tuberosidade e, no plano coronal, são corrigidos o alinhamento em varo, a translação da tuberosidade e o espessamento da parede lateral.

A redução do corpo do calcâneo deve realinhar os componentes da fratura com uma redução funcional indireta, que pode ser alcançada por meios fechados. A estabilidade relativa é adequada para a fixação do corpo.

Na maioria dos casos, abordagens laterais são usadas para esse propósito. A abordagem estendida lateral em “L” permite a redução aberta das articulações subtalar e calcaneocuboide e do corpo do calcâneo, enquanto a abordagem minimamente invasiva, a do seio do tarso, permite a redução aberta das articulações subtalar e calcaneocuboide e a redução fechada do corpo.

Prather et al., 27 em um estudo experimental com cadáveres, compararam a abordagem estendida com a abordagem do seio do tarso e demonstraram que ambas fornecem área de exposição articular equivalente, embora a versão estendida permita melhor visualização da parede lateral.

## Abordagem Cirúrgica

### Seio do Tarso

A abordagem do seio do tarso minimiza os danos aos tecidos moles circundantes, reduzindo o risco de deiscência e infecção. 28 29 30

Yao et al., 30 em uma revisão sistemática e meta-análise de 12 estudos, compararam complicações da ferida e qualidade da redução entre pacientes com fraturas de calcâneo tratados com as abordagens do seio do tarso e estendida. Os autores demonstraram que o grupo do seio do tarso apresentou menor incidência de complicações da ferida e qualidade de redução comparável.

Esta abordagem fornece reduções comparáveis às da abordagem lateral estendida em “L”, com desfechos funcionais semelhantes, mas menos complicações. No entanto, a técnica do seio do tarso é mais complexa e requer uma curva de aprendizado maior. 5 31 32 33 34

Com o paciente em decúbito lateral, a incisão começa posterior ao maléolo lateral e se estende em direção à base do quarto metatarsal. O comprimento depende do acometimento da fratura da articulação calcaneocuboide. A dissecção subcutânea atinge o músculo extensor curto dos dedos, que é refletido distalmente para expor o calcâneo anterior. Deve-se ter cuidado com os ramos sensíveis do nervo sural.

A dissecção no ângulo de Gissane expõe o seio do tarso, permitindo a visualização dos ligamentos interósseos e da articulação subtalar posterior. Os tendões fibulares são movidos posteriormente para aumento da exposição. Se necessário, os ligamentos interósseos fibulocalcâneos e subtalares são liberados.

**Fig. 3:**
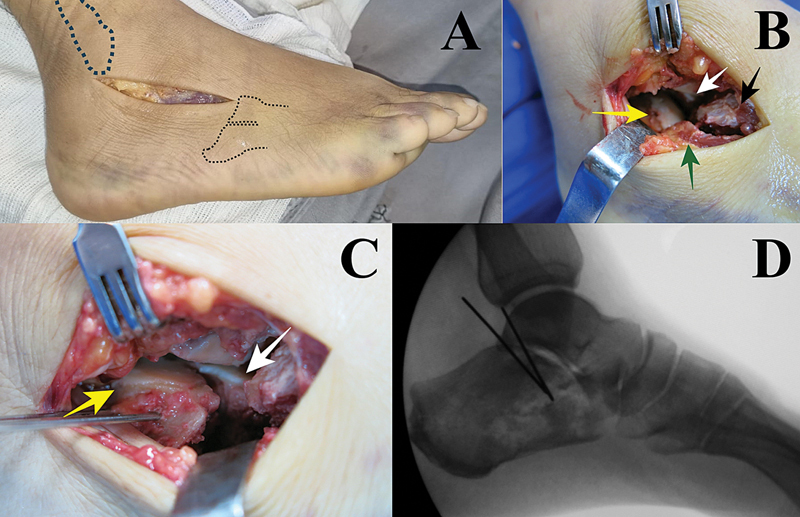
Abordagem do seio do tarso. ( **A** ) Parâmetros da incisão. ( **B** ) Visualização dos fragmentos pela incisão. Seta amarela – depressão da articulação subtalar; seta branca – fragmento constante; seta preta – porção anterior do calcâneo; seta verde – parede lateral. ( **C** ) Redução direta da articulação subtalar. Seta amarela – depressão da articulação subtalar; seta branca – fragmento constante. ( **D** ) Fixação temporária com fios de Kirshner.

Plantar à borda inferior da incisão, sob os tendões fibulares, com dissecção romba, ganha-se acesso à parede lateral do calcâneo e, posteriormente, à tuberosidade. Portanto, a abordagem do seio do tarso permite a visualização direta das articulações subtalar posterior e calcaneocuboide, da parte superior da parede lateral do calcâneo e dos tendões fibulares ( Fig. 3 ).

A manobra de Essex-Lopresti, 22 visualizada pela abordagem do seio do tarso, é particularmente eficaz para fraturas de língua. Nesses casos, o fragmento lateral fraturado da articulação subtalar posterior permanece preso à tuberosidade. O objetivo da manobra é elevar o fragmento articular empurrando a tuberosidade em sentido distal. A abordagem do seio do tarso permite a visualização direta da redução anatômica do fragmento subtalar lateral em alinhamento com o fragmento constante medial.

O procedimento começa pela flexão plantar do tornozelo e uso de um fio de Steinmann ou pino de Schanz para empurrar a tuberosidade calcânea em direção distal. Instrumentos rombos auxiliam a obtenção de uma redução precisa da articulação. Uma vez obtida a redução, a fixação temporária com fios de Kirschner garante o alinhamento anatômico da articulação e a redução funcional do corpo do calcâneo. A fixação definitiva é, então, realizada com parafusos de tração para a articulação subtalar e parafusos de posicionamento para a tuberosidade.

As fraturas por depressão, que são mais cominutivas e de redução mais difícil, exigem a liberação de fragmentos ósseos da articulação e do corpo do calcâneo para criar espaço para o alinhamento. 2 A redução começa com a manipulação do calcâneo anterior para restaurar a articulação calcaneocuboide, estabelecendo mais um parâmetro para redução com o sustentáculo do tálus.

Os fragmentos da articulação subtalar posterior são, então, elevados proximalmente usando instrumentos rombos e alinhados aos remanescentes da articulação medial no sustentáculo do tálus. A tuberosidade é reposicionada pela introdução de um instrumento rombo lateralmente, abaixo do sustentáculo do tálus, forçando a translação medial para centralização da tuberosidade sob o eixo de carga tibial. Um fio de Steinmann ou pino de Schanz serve como um *joystick* para guiar a tuberosidade em sentido posterior e plantar, aumentando o comprimento e a altura do calcâneo. A rotação coronal também é corrigida durante esse processo, reduzindo o alinhamento em varo.

Com a redução do fragmento completa, a parede lateral é empurrada em sentido medial para correção do espessamento. A fixação temporária com fios de Kirschner é verificada à fluoroscopia e seguida pela fixação definitiva ( Fig. 3 ).

A estratégia ideal de fixação visa a estabilidade absoluta das articulações subtalar e calcaneocuboide utilizando parafusos de tração ancorados ao sustentáculo do tálus. Entretanto, em casos com cominuição significativa, atingir a estabilidade absoluta é um desafio, com necessidade de parafusos de posicionamento para manutenção da redução anatômica.

A tuberosidade posterior, a parede lateral e a Porção anterior do calcâneo são fixados com estabilidade relativa, obtida com parafusos ou placas, dependendo da morfologia e cominuição da fratura.

Discutindo opções de síntese nestas situações, Ni et al. 35 (2016) demonstraram que a estabilidade mecânica de fraturas fixadas com placas de bloqueio é comparável àquela conseguida apenas com parafusos. Estudos subsequentes confirmaram a semelhança de rigidez, função pós-operatória e desfechos de reabilitação entre parafusos e placas. 25 36 37 Portanto, a fixação por parafuso é preferida na maioria dos casos devido à menor lesão do envelope de partes moles e menor custo.

**Fig. 4:**
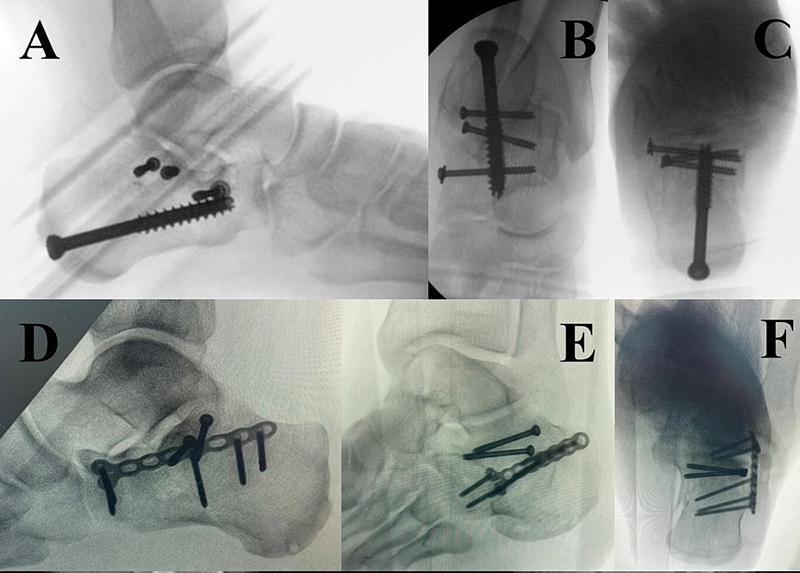
Fixação com parafusos e placa. ( **A–C** ) Fixação com parafusos, reduzindo todos os componentes da fratura do calcâneo. ( **D–F** ) Porção anterior cominuída do calcâneo. Fixação da articulação subtalar com parafusos e do calcâneo com placa bloqueada.

A manutenção da redução da tuberosidade é feita com um parafuso de posicionamento fixado na porção anterior do calcâneo. O parafuso pode ser direcionado de posterior para anterior ou de dorsal e posterior para anterior e inferior, dependendo da qualidade óssea adequada para a conservação da fixação do parafuso 12 20 38 ( Fig. 4 ).

Quando a fragmentação e a ausência de massa óssea impedem a fixação estável do parafuso, placas de bloqueio são usadas para estabilizar as reduções entre os elementos calcâneos anteriores e posteriores. 2 Placas específicas projetadas para a abordagem do seio do tarso ou placas de bloqueio calcâneo convencionais podem ser moldadas para bom posicionamento ( Fig. 4 ). A haste calcânea é uma opção de fixação atual, seguindo os mesmos conceitos descritos anteriormente, com resultados iniciais animadores, proporcionando estabilidade comparável à da placa de bloqueio. Entretanto, mais estudos clínicos são necessários para entender em quais situações seu uso traz mais benefícios. 39

Em casos de cominuição extensa da articulação e do corpo, em que a fixação entre os fragmentos é inviável, mesmo com placas, pode-se realizar a artrodese primária da articulação subtalar. Essa abordagem mantém o formato do calcâneo e o alinhamento do retropé. 6

## Abordagem Lateral Estendida

Entender a anatomia vascular é fundamental para planejar abordagens cirúrgicas de maneira eficaz.

**Fig. 5:**
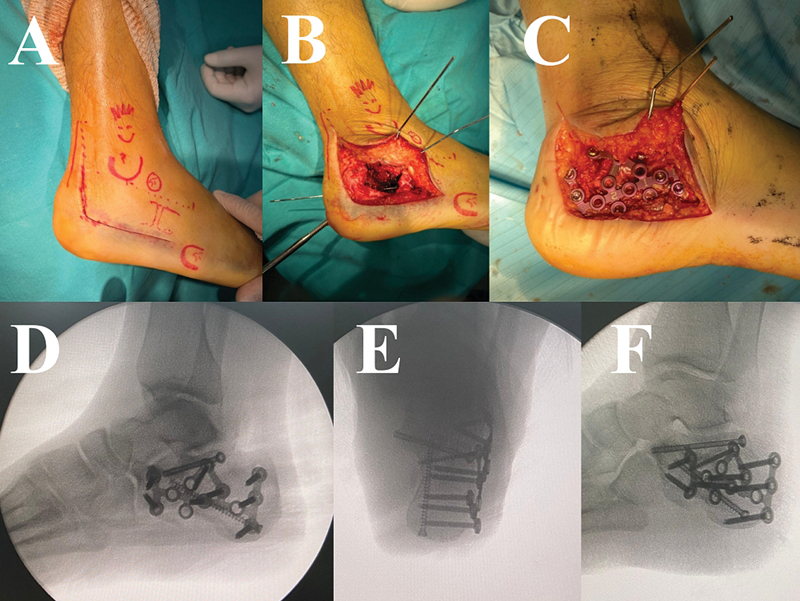
Abordagem lateral estendida. ( **A** ) Incisão lateral em formato de “L”. ( **B** ) Schanz lateralmente para manipulação da tuberosidade. ( **C** ) Placa calcânea. ( **D** ) Projeção fluoroscópica em perfil. ( **E** ) Projeção fluoroscópica axial. ( **F** ) Projeção fluoroscópica de Broden.

Nesta abordagem, descrita por Benirschke 40 em 1993, o paciente é posicionado em decúbito lateral e uma incisão lateral em formato de “L” é realizada ( Fig. 5A ). Os tecidos moles profundos são incisados precisamente ao longo da incisão cutânea e dissecados juntos em um único plano até o periósteo da parede lateral. A porção vertical começa 2 cm proximal à ponta do maléolo lateral, entre o terço posterior da fíbula e o terço anterior do tendão calcâneo, com o nervo sural e a artéria calcânea lateral localizados anteriormente. A porção horizontal entre a pele dorsal e plantar, demarcada pela compressão do calcanhar, estendendo-se até a base do quinto metatarsal. As duas porções da incisão se encontram em um ângulo obtuso para minimizar o risco de necrose no ápice. 2

O retalho é retraído suavemente durante a dissecção subperiorteal ao longo da parede lateral até a ponta da fíbula. O retalho inteiro é elevado como uma unidade e mantido com dois fios de Kirschner, um na fíbula e outro no colo do tálus. O retalho não volta a ser manipulado durante o restante do procedimento.

Esta abordagem permite 74% de visualização da articulação subtalar (semelhante à abordagem do seio do tarso), 71% da parede lateral e 3% da tuberosidade anterior. 27

O fragmento articular superolateral impactado da parede lateral é cuidadosamente elevado e colocado em solução salina na mesa auxiliar. Dois fios de Kirschner são inseridos a partir da tuberosidade posterior sem cruzar a linha de fratura. A manipulação da tuberosidade posterior é realizada usando um pino de Schanz de 4,5 mm, colocado em sentido lateral para medial como descrito por Benirschke, 40 ou posteriormente, como indicado por Rammelt and Zwipp 1 ( Fig. 5B ).

A manipulação é feita nesta sequência: tração para restaurar o comprimento, translação medial e, então, translação lateral para atingir o valgo fisiológico. Uma vez obtida a posição correta, os fios de Kirschner previamente colocados são avançados da tuberosidade para o fragmento sustentacular, alcançando estabilização temporária e restauração da parede medial.

A redução da articulação subtalar é realizada de medial para lateral sob visualização direta, projeções de Broden ou artroscopia seca. A compressão é obtida com um ou dois parafusos. A tuberosidade é alinhada à articulação reduzida, a altura e o alinhamento varo-valgo são controlados e a fixação é concluída com pinos. O processo anterior é reconstruído de medial para lateral, usando o cuboide como guia. 2

Por fim, a parede lateral é reposicionada e fixada com uma placa calcânea periarticular, fixada com parafusos na tuberosidade posterior, região subtalar e processo anterior. A placa auxilia a manutenção do eixo calcâneo ( Fig. 5C ).

## Período pós-operatório

O cuidado pós-operatório se concentra na evolução do envelope de partes moles. Um curativo levemente compressivo é aplicado após a cirurgia para controle do sangramento. Uma órtese removível dá suporte à mobilidade precoce e previne a deformidade em equino.

Após 10 a 14 dias, as suturas são removidas e os esforços para melhorar a amplitude de movimento e a força são intensificados. Em 10 semanas, começa a sustentação parcial progressiva de peso com a órtese. Em 12 semanas, com evidências clínicas e radiográficas de consolidação, a sustentação total do peso em calçados de sola firme é permitida. O acompanhamento radiográfico é realizado periodicamente.

O momento de iniciar a descarga de peso ainda é motivo de debate, embora a maioria dos autores o adie. Chongmuenwai e Thitirangsi 12 avaliaram um protocolo de sustentação de peso pós-operatório precoce. Os autores compararam o início da sustentação de peso parcial progressiva conforme tolerado às 4 ou 8 semanas pós-operatórias e não observaram diferenças na manutenção da redução.

## Complicações

As complicações comuns das fraturas do calcâneo incluem osteoartrite pós-traumática, lesões neurológicas, deiscência de sutura, infecções, não consolidação, má consolidação e tendinite fibular. 4

## Conclusão

As fraturas do calcâneo impactam significativamente a vida dos pacientes, muitas vezes levando a desafios sociais e ocupacionais. A compreensão completa da anatomia do osso e dos padrões de fratura é essencial para atingir a redução ideal. Este processo começa com a liberação dos fragmentos da fratura para permitir o posicionamento anatômico preciso.

A intervenção cirúrgica por meio da abordagem do seio do tarso oferece desfechos funcionais comparáveis aos da abordagem lateral estendida em “L”, ao mesmo tempo em que reduz complicações de partes moles. Apesar da curva de aprendizado mais longa, essa abordagem deve ser fortemente considerada.

De modo geral, as fixações articular e do corpo do calcâneo podem ser obtidas com parafusos, desde que haja um fragmento constante de tamanho adequado e densidade óssea suficiente. Os parafusos produzem resultados comparáveis aos das placas bloqueadas, com redução de lesão do envelope de partes moles e custo.

As placas de bloqueio são indicadas caso a cominuição grave impeça a fixação estável do parafuso. Em casos de cominuição extensa da articulação e do corpo, a artrodese primária da articulação subtalar pode garantir estabilidade do calcâneo e do retropé.
